# Prevalence of early childhood caries and enamel defects in four and five-year old Qatari preschool children

**DOI:** 10.1186/s12903-016-0267-z

**Published:** 2016-08-18

**Authors:** Asmaa Alkhtib, Aghareed Ghanim, Meredith Temple-Smith, Louise Brearley Messer, Marie Pirotta, Michael Morgan

**Affiliations:** 1 Primary Health Care Corporation, Qatar, University of Melbourne, Melbourne, VIC Australia; 2 Melbourne Dental School, University of Melbourne, Melbourne, VIC Australia; 3 Department of General Practice, University of Melbourne, Melbourne, VIC Australia; 4 Oral Health CRC, University of Melbourne, Melbourne, VIC Australia; 5 Oral Health Division, Primary Health Care Corporation, PO Box 26555, Doha, Qatar

**Keywords:** ECC, s-ECC, Developmental defects of enamel, Primary teeth, Epidemiology, Children, Qatar

## Abstract

**Background:**

Dental caries is the most prevalent chronic disease in early childhood in most communities worldwide. Several studies conducted in the Gulf Cooperation Council countries have documented a high prevalence of early childhood caries (ECC). To date, no studies have been conducted in Qatar to examine the prevalence of ECC and enamel developmental defects in preschool children. The present study aimed to determine the prevalence of ECC and enamel defects in preschool children aged four and five years attending kindergartens in Qatar.

**Methods:**

A descriptive cross-sectional study was performed among 250 children randomly selected from 16 public kindergartens. Caries experience was measured using the World Health Organization caries criteria (dmft) for tooth rather than surface; enamel defects were scored by the modified developmental defects of enamel index. Descriptive statistics and Chi-Square test of relatedness were used.

**Results:**

A response rate of 63 % was reported. The overall caries prevalence of the study sample was 89.2 %; 15.6 % of the examined children had ECC and 73.6 % had severe ECC. Developmental enamel defects were present in 39 % of children and 27 % had demarcated enamel opacities.

**Conclusion:**

The high rate of dental caries and enamel defects recorded in this study for this young age group has strongly emphasized the necessity of community-based preventive programs and professional care that should begin during pregnancy and in early childhood.

## Background

Qatar is a country of over two million people located in the Arabian Gulf region, which, together with another five countries, form the Gulf Cooperation Council (GCC). Recent development in social and dietary changes has resulted in lifestyle-related diseases such as diabetes, cardiovascular disease and dental caries [1]. Whilst some health programs are available in Qatar to address these conditions in adults, the need for such programs has not yet been demonstrated in children.

Dental caries affecting children 71 months or younger is referred to as early childhood caries (ECC), and this condition can develop as early as the eruption of the primary teeth [2]. The disease is diagnosed as Severe Early Childhood Caries (s‐ECC) in children aged three to five years, where there is one or more decayed, filled or missing (due to caries) lesions in a maxillary anterior tooth or dmft of ≥ 4 (at age 3), ≥ 5 (at age 4), ≥ 6 (at age 5)” [3].

It is known that dental caries can affect the general health and wellbeing of affected individuals, especially children. Many studies have revealed the negative impact of dental caries on the quality of life of children ranging from being ashamed to smile and speak, to difficulty in eating and malnutrition [4–7]. Further to this, research proves that untreated tooth decay in children causes pain and infection that leads to more missed school days and lower academic performance compared to students who have good oral health [8, 9].

Whilst it is recognised that dental caries is caused by a multiplicity of factors including frequent consumption of fermentable carbohydrates, a less well recognised cause may be that the presence of enamel defects contributes to increased caries risk by making teeth more susceptible to post-eruptive breakdown and subsequent lesion development [10–12]. A defective tooth surface may provide a suitable site for the adhesion and colonisation of cariogenic bacteria, and due to cleaning difficulties may cause the bacteria to be retained at the base of the defect resulting in a more rapidly developing carious lesion than otherwise would occur on a sound tooth surface [13, 14].

Developmental defects of enamel (DDE) are variations in quality and quantity of the enamel, resulting from disturbances in amelogenesis [15, 16]. Enamel hypomineralisation is a qualitative defect presenting alterations in enamel translucency and opacity. The defective enamel is of normal thickness and opacities can be diffuse or demarcated with white, yellow, or brown colour. The enamel is soft and porous and can chip off easily, leading to unprotected dentine and also an unexpectedly rapid caries development. On the other hand, Enamel hypoplasia is a quantitative enamel deficiency that may present as pits, grooves, or generalised lack of surface enamel [15, 16].

Dental caries is a disease which is totally preventable with simple measures including appropriate diet and good oral hygiene [17]. However, dental caries remains the most prevalent chronic disease in early childhood [18]. It can be seen from Table 1 that studies on the prevalence of ECC globally and regionally reported great variation. While developed countries have low prevalence, countries in the GCC and the Middle East region demonstrate high prevalence of ECC [19–26].

**Table 1:** Prevalence and severity of dental caries in some developing and developed countries

Place, (Study)	Sample = N	Caries Prevalence	Caries severity
	(Age in years)		
Saudi Arabia (Al-Malik et al., 2001) [19]	987 (2–5)	73 %	34 % had rampant caries
Amman, Jordan, (Sayegh et al., 2005) [20]	1,140 (4–5)	67 %	31 % had dmft >4
Al Ain, UAE (Al-Hosani and Rugg-Gunn, 1998) [21]	640 (2,4,5)	2yo = 32 %	Mean dmft =7.6
		4yo = 77 %	
		5yo = 89 %	
Ajman, UAE (Hashim et al., 2006) [22]	1,297 (5–6)	76 %	Mean dmft = 4.4 (4.3)
Kosovo (Begzati et al., 2010) [23]	1,008 (1–6)	86 %	Mean dmft = 5.8
Australia (Centre for Oral Health Strategy, 2009) [24]	7,975 (5–12), 981 (5)	40 %	dmft = 1.6
New Hampshire USA (Anderson et al., 2010) [25]	607 (3–5)	40 %	30 % had dmft >1
UK (Davies et al., 2011) [26]			
Scotland	12,442 (5)	29-48 %	dmft = 1.2- 2.1
England	139,727	31 %	dmft = 1.1
Wales	12,662	-	dmft = 2.0

*dmft* Number of decayed, missing and filled primary teeth

Similarly, the distribution of DDE in primary dentition has been investigated in different parts of the world with prevalence varying between 4 % and 75 %, depending on the population studied and scoring criteria [11, 27, 28]. It has been shown that developmental disturbances of the enamel in the primary dentition may be predictive of similar alterations in the permanent dentition [11, 27–30].

To date, the prevalence of dental caries and DDE in preschool children in Qatar has not been reported. This study aimed to conduct an assessment of early childhood caries and enamel defects in preschool children aged four to five years attending kindergartens in Qatar, using a cross-sectional design.

## Methods

### Population and sample

The present study represents part of a broader study examining strategies on the implementation of a primary care oral health promotion project. The sampling for this study aimed to be representative of children attending governmental kindergartens in Qatar. Based on relevant literature the sample size required for this study was estimated to be 291 subjects [31]. Given the high prevalence figure of ECC in the GCC region the estimated prevalence of dental caries in Qatar would not be less than 50 % and the marginal error was set at 2.5 %; 400 subjects were approached in order to reach the required sample size. For the present study a two-stage stratified clustered random sampling method was implemented. The sampling method identified the clusters randomly at the first stage then conducted independent simple random sampling for each cluster.

The sample was identified through the Department of Early Childhood Education in Qatar, which provided a list of 32 governmental kindergartens registered in the school year 2011/2012 with about 6000 children registered. Sixteen kindergartens (50 % of the total) were selected using a random number generation software (Excel 2007 (v12.0), Microsoft Corporation, Seattle, Washington, USA) and each kindergarten was considered as a cluster stratum. A total of 30–35 children were chosen randomly from different classes in each kindergarten. The age range in all kindergartens in Qatar is 4–5 years.

The inclusion criteria were the child being healthy and being a Qatari national attending a government kindergarten. The exclusion criteria included children in whom there was significant dental anxiety and who refused to have their teeth examined and children who have been ideintified by the kindergarten nurse to have medical or intellectual problems.  Data collection occurred in 2011–2012 over a period of 4 weeks. The public water supply in Qatar is not fluoridated.

### Clinical examination and assessment criteria

An informed consent was requested and obtained from parents. All participating children had their teeth examined within the kindergarten premises, with the child laying down either on a table or a mattress on the floor. Examination was performed by two examiners (AA, AI) using a disposable dental mouth mirror with inbuilt fibre optic light (Denlite, Miltex Inc, Yourk, PA, USA); cotton rolls were used to clean and dry the teeth where necessary. Caries experience was assessed using the decayed, missing and filled teeth index (dmft) and scored according to WHO standard criteria [32]. No radiographs were taken. Enamel defects were assessed according to the modified DDE Index [16]. The scoring was made on the tooth level recording the most severe lesion. An enamel defect of one millimetre or less was considered as sound [16]. The data were recorded onto two specially prepared dental record charts by a trained research assistant.

Training and calibration exercises for the examiners in diagnosing DDE and dental caries were undertaken with the aid of an experienced epidemiologist at The Melbourne Dental School using a set of 20 photographs three times for each DDE and dental caries scoring codes. Using the Kappa statistic, the intra-examiner agreements for DDE and dental caries scores were 0.76 and 0.79, respectively. The inter-examiner agreements for DDE were 0.82 and 0.77 for dental caries [33].

Statistical analysis of the data was performed using the Statistical Package for the Social Sciences (SPSS Incorporated, Chicago, Illinois, Version 20.0, USA). Descriptive statistics and frequency tables for dependent variables (dental caries and DDE) and independent variables (age and gender) were prepared. The outcome measures were the presence of caries (dmft ≥1) and the presence of enamel defects. The Pearson Chi-Square test of relatedness (*χ*^2^ test) was used to compare the proportions. Continuous variables were compared using the one-way analysis of variance (ANOVA) test. The critical level for alpha was set at 0.05.

## Results

Of 400 families contacted, 250 gave consent for clinical examination (response rate 63 %). A total of 250, four to five-year old preschool children with a mean age of 4.4 years (±0.4), were examined for dental caries and developmental enamel defects, with a distribution of 127 (51 %) males and 123 (49 %) females. The number of children selected from each kindergarten ranged from 13–26 children, according to attendance of the children on the day of the clinical examination.

The overall caries prevalence of the study sample was 89.2 % (*n* = 223); 15.6 % (*n* = 39) of the children had ECC and 73.6 % (n = 184) had s-ECC. No significant difference in the prevalence of ECC and s-ECC was found by comparison of age and gender (Table 2).

**Table 2:** Numbers and percentages of children with no detected caries and those with rampant caries by gender

Children with	Male N (%)	Female N (%)	Total N (%)
No caries detected	13 (10.2)	14 (11.4)	27 (10.8)
ECC	13 (10.3)	26 (21.1)	39 (15.6)
s-ECC	101 (79.5)	83 (67.5)	184 (73.6)

*ECC* Early Childhood Caries; *s-ECC* Severe Early Childhood Caries

The mean (± SD) caries experience of the total sample was 7.6 (±5.1). The mean dmft in the group with ECC and s-ECC were 3.3 (±2.1) and 4.8 (±4.7), respectively. The mean caries experience in the four-year old group was 7.6 (±5.2), which was similar to the five-year old children, 7.6 (±5.1). Males and females demonstrated similar dmft scores (7.5 (±4.9) and 7.6 (±5.3), respectively). In the entire sample there were no significant differences in the dental caries prevalence by comparison of age or gender.

Analysis of the distribution of dental caries by dental arch revealed that dental caries was significantly more likely in maxillary teeth than mandibular teeth (4.6 (±3.4) vs. 3.3 (±2.4); F ratio (1,249) =30.1, *p* = 0.001). Analysis by mouth side revealed that the left side had significantly higher caries experience than the right side (3.9 (±2.8) vs. 2.9 (±1.8); F ratio (1,249) =78.6, *p* = 0.001) (Table 3). However, no significant differences were seen between caries experience in the maxillary versus mandibular arch and mouth side, when analysed according to age and gender.

**Table 3:** Mean dmft and its components for the total sample by gender, age, arch type and side of the mouth

Mean ± Standard Deviation				
	dmft	dt	mt	ft
Gender				
Male	7.6 ± 5.0	7.0 ± 4.4	0.3 ± 0.8	0.3 ± 1.0
Female	7.6 ± 5.4	7.1 ± 5.3	0.3 ± 0.8	0.3 ± 0.7
Total	7.6 ± 5.2	7.1 ± 5.0	0.3 ± 0.8	0.3 ± 0.8
Age (years)				
4	7.6 ± 5.2	7.8 ± 5.1	0.3 ± 0.8	0.2 ± 0.6
5	7.6 ± 5.1	7.0 ± 5.0	0.3 ± 0.9	0.4 ± 1.0
Dental arch				
Maxillary	^a^4.6 ± 3.4	4.3 ± 3.2	0.2 ± 0.8	0.1 ± 0.7
Mandibular	3.3 ± 2.4	3.0 ± 2.3	0.2 ± 0.6	0.8 ± 0.6
Arch side				
Right	4.0 ± 1.8	3.0 ± 1.7	0.2 ± 0.6	0.1 ± 0.4
Left	^b^4.0 ± 2.8	3.7 ± 2.7	0.2 ± 0.5	0.1 ± 0.5

^a^,^b^Statistically significant differences between maxillary and mandibular dental arches as well as between the right and left mouth sides in the dmft experience at *p* < 0.001, One Way ANOVA test

The pattern of dmft distribution by tooth type represented with the second molars as the most affected teeth (2.6 ± 1.5), followed by the first molars (2.1 ± 1.6) and central incisors (1.3 ± 1.3), whilst canines were the least affected (0.9 ± 1.2). No statistical significant difference was found in the mean number of affected teeth per tooth type by gender.

**Fig. 1:**
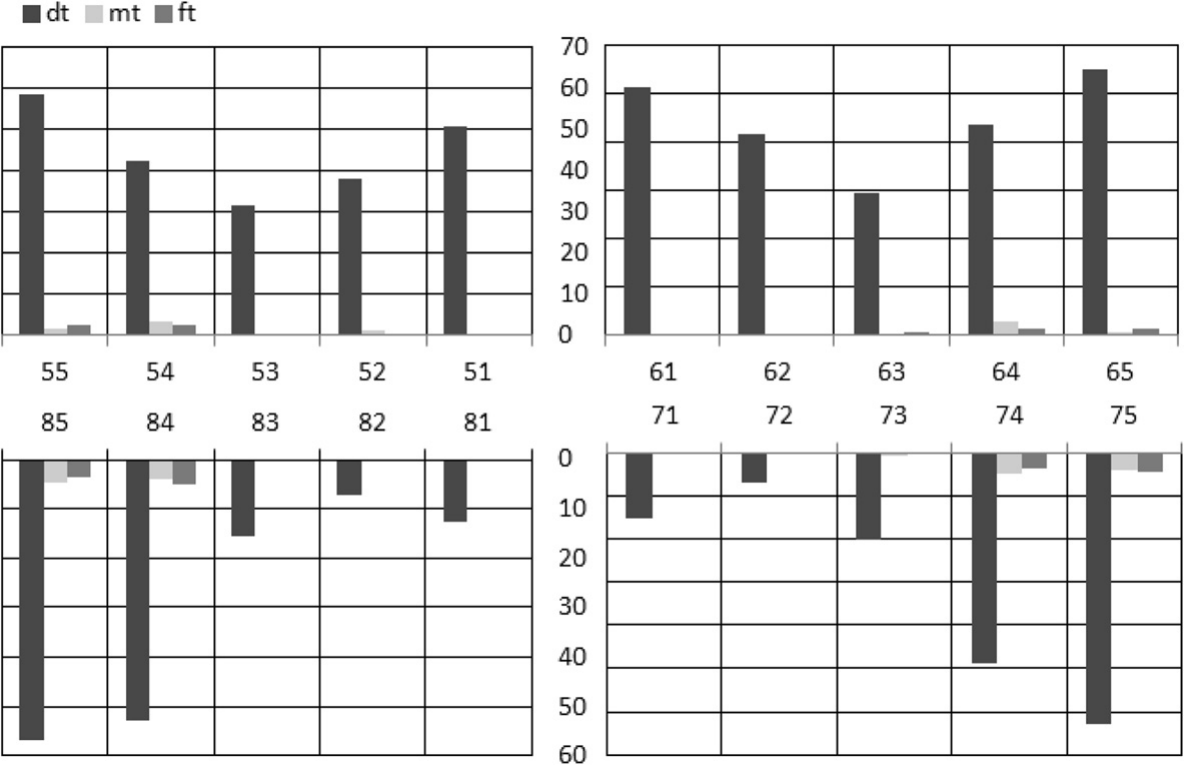
Percentage of decayed, missing and filled teeth for the total sample

The percentage distribution of the dt, mt and ft components are shown in Fig. 1. For the dt component, in general, the second molars showed the highest score whereas the maxillary anterior teeth were affected as severely as that for the first primary molars. Maxillary canines were next in sequence whilst the mandibular anterior teeth were the least affected. The distribution of mt and ft components revealed that the molars were almost the only teeth involved. No statistical significant difference was presented in any of the dmft components by gender.

**Fig. 2:**
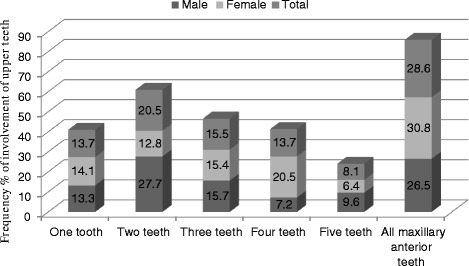
Dental caries experience by frequency of involvement of maxillary anterior teeth

The distribution of dental caries experience by frequency of involvement of maxillary anterior teeth is shown in Fig. 2. Over twenty eight  of the affected children had all maxillary anterior teeth affected, 20.5 % had two teeth affected, 15.5 % had three teeth, one tooth - or four teeth were affected in 13.7 %, and 8.1 % had five teeth affected. The trend towards children to have four teeth affected was more apparent in girls than boys; however, no significant difference was found in the number of carious teeth by gender.

Twenty-seven percent of the children had demarcated enamel opacities while diffuse enamel opacity and hypoplasia affected 9 % and 3 %, respectively. On a tooth level, of the 4,930 teeth examined, 229 teeth were affected by DDE (tooth prevalence 4.6 %). The second primary molar was the tooth most affected (26.6 %, *n* = 61) followed by central incisors (24.9 %, *n* = 57); canines and first primary molars were affected similarly (19.2 %, *n* = 44) whilst lateral incisors were the least frequently affected teeth (9.6 %, *n* = 22). In the entire sample there were no significant differences in the prevalence of DDE by comparison of age or gender (Table 4). The enamel defects did not have a statistically significant association with the occurrence of dental caries [*χ*^2^ (1) = 1.6, *p* = 0.3].

**Table 4:** Distribution of developmental defects of enamel (DDE) by age and gender

	Age (years)			Gender		
	4	5	Total	Male	Female	Total
	N (%)	N (%)	N (%)	N (%)	N (%)	N (%)
DDE						
DDE-free	80 (64.0)	73 (58.4)	153 (61.2)	77 (60.6)	76 (61.8)	153 (61.2)
Demarcated opacities	31 (24.8)	36 (28.8)	67 (26.8)	32 (25.2)	35 (28.5)	67 (26.8)
Diffuse opacities	10 (8.0)	13 (10.4)	23 (9.2)	15 (11.8)	8 (6.5)	23 (9.2)
Hypoplasia	4 (3.2)	3 (2.4)	7 (2.8)	3 (2.4)	4 (3.2)	7 (2.8)

## Discussion

The present study documented widespread neglect of the oral health of preschool children in Qatar. Untreated decayed teeth dominated the dmft score among the children in this study, indicating a high rate of unmet treatment needs. Additionally, this study provided information on the caries status of preschool children in age groups not included in the national surveys. Caries prevalence of the examined population (89 %) was comparable to other studies conducted in regions with socio-behavioural characteristics similar to that of the Qatar region. In Saudi Arabia and United Arab Emirates a high caries prevalence in preschool children was also found (75 % and 70–80 %, respectively) [22, 34, 35]. According to a national epidemiologic survey conducted recently in Kuwait, kindergarten children who are caries-free at the age of 4–5 years do not represent more than 24–32 % of their population [36]. These findings are higher than what we found here in Qatari children (10.8 %). This figure is still significantly lower than figures published by many developed countries such as the United Kingdom, Australia and Sweden where 40–60 % of 5 year-old, 66 % of 4 to 6 year-old and 69 % of 3 year-old children, respectively, were free of dental caries [24, 26, 37]. A possible explanation for this considerable difference between developed and developing countries for this age group could be attributed to the lack of an effective fluoridation policy, an inadequate oral healthcare system, differing dietary habits including high consumption of refined sugars by children, and the absence of oral health awareness among parents in developing countries.

The prevalence of DDE in the present study (39 %) lies within the ranges previously reported for children in other countries, [38–40] however the study did not find any statistically significant association between the occurrence of DDE and dental caries experience. This is in contrast to findings of previous studies which showed a clear association between DDE and caries risk by increasing the susceptibility to breakdown and subsequent cariogenic attack [10–14]. The high caries trends displayed in the present study could have masked many DDE lesions and render the reported prevalence of the enamel defects lower than the true figure. In particular, the diagnosis of tooth substance loss because of caries as a primary cause was considered pre-eminently, which may underestimate the true number of teeth with breakdown because of DDE as a primary cause.

The findings of the present study show a significant oral morbidity in this young age group, despite the preventable nature of dental caries. The high rate of unmet treatment needs observed among preschool children may reflect a lack of community awareness and understanding that prevention and treatment of caries should begin in early childhood, and parental indifference in association with belief that the primary teeth are replaceable by permanent teeth. Early intervention with preventive measures could play a significant role in preventing dental caries in preschool children [41]. There is a clear need for community-based preventive programs and professional care that should begin during pregnancy and in early childhood.

Opportunities to deliver such preventive measures in the Qatari health system include oral health education of pregnant mothers and mothers of children attending the vaccination services in the first two years of life. The incorporation of oral health screening with the periodic general health and development screening of children from 12 months to four years could be performed routinely [41].

This field study had several limitations. Radiographs were not taken for ethical reasons and technical difficulties. Comprehensive dental histories were unavailable at examination, as parents were not present. Data on missing anterior teeth for reasons other than caries were excluded, assuming children at this age group may have early exfoliation of primary incisors. However, it is possible that some anterior teeth were missing as a result of trauma.

The response rate for participation was relatively low for the dental examination. This may be as a result of no previous experience of parents in allowing their children to participate in epidemiological studies in Qatar. Also, parents may have feared their child may have a negative experience and especially when they were not present during the clinical examination. However, the plain language statement that was provided to the parents with the consent included clear and comprehensive information to fully inform parents with the friendly circumstances of the dental examination.

There is a possibility of selection bias as the sample was drawn from government kindergartens, and children not attending kindergarten at all or attending private kindergartens may have shown different results from the present sample.

Furthermore, the sample size was estimated to be 291 children, but, as reported in the result section, only 250 parents gave consent for their children to be examined. The total number of children in governmental KG was around 6000 children and the sampled children (*n* = 250) represented about 4 % of eligible population at the time of the study. With the high prevalence of dental caries in the 250 children, the 40 children difference may not have shown substantial different results. However, a comprehensive national survey with a sample representative of all children in Qatar is needed, to fully assess the problem of ECC and DDE in Qatar. Despite all these limitations, this study provides a foundation for a comprehensive oral health policy for young children under current proposal to health authorities in Qatar.^1^

## Conclusion

The overall prevalence of dental caries in this sample of preschool Qatari children was 89 %; ECC and s-ECC comprised 15.6 % and 73.6 %, respectively. Enamel defects were predominantly demarcated opacities (27 %), with other lesions including diffuse opacities and hypoplasia affecting 12 % of children. The proportion of males and females affected by dental caries and DDE were similar with no statistical significant difference. An early intervention program is urgently needed to help control ECC and other dental defects in Qatar.

## Abbreviation

DDE, Developmental defects of enamel; ECC, early childhood caries; GCC, Gulf Cooperation Council; N, Number, UAE: The United Arab Emirates; s-ECC, Severe Early Childhood Caries; SPSS, Statistical Package for Social Sciences; UK, The United Kingdom; USA, The United States of America; WHO, World Health Organization, dmft: decayed, missing, filled teeth

## Footnotes

1 https://www.moph.gov.qa/health-features/qatar-health-issue-07/working-together-to-keep-qatar -smiling#.Vziv6bLvRTc.google
